# Equilibrative Nucleoside Transporters-1 Inhibitors Act as Anti-epileptic Agents by Inhibiting Glutamatergic Transmission

**DOI:** 10.3389/fnins.2020.610898

**Published:** 2020-12-17

**Authors:** Shih-Yin Ho, I-Chun Chen, Kai-Chieh Chang, Hsiao-Ru Lin, Che-Wen Tsai, Chun-Jung Lin, Horng-Huei Liou

**Affiliations:** ^1^Department of Neurology, National Taiwan University Hospital, College of Medicine, National Taiwan University, Taipei, Taiwan; ^2^Department of Pharmacology, College of Medicine, National Taiwan University, Taipei, Taiwan; ^3^National Taiwan University Hospital Yunlin Branch, Douliu, Taiwan; ^4^School of Pharmacy, National Taiwan University, Taipei, Taiwan

**Keywords:** adenosine, ENT-1, maximal electroshock seizure, pentylenetetrazol-induced seizure, whole-cell patch-clamp recording

## Abstract

**Background and Purpose:** Adenosine dysregulation is associated with the occurrence of the epilepsy and equilibrative nucleoside transporters-1 (ENT-1) functions as an important regulator of extracellular adenosine in the brain. This study was aimed to prove the anti-epileptic effect of BBB permeable ENT-1 inhibitors, JMF1907 and J4, on animal models of various epilepsy, and the mechanisms that are involved.

**Experimental Approach:** Maximal electroshock seizure (MES), pentylenetetrazol (PTZ)-induced seizure and kindling models were used as mouse models of generalized tonic-clonic epilepsy, generalized myoclonic epilepsy, and partial epilepsy, respectively. The epilepsy frequency, duration, and Racine score were evaluated. Whole-cell recordings were made from the hippocampal dentate granule cells by using a patch-clamp technique in the brain slice of the mice.

**Key Results:** In MES, JMF1907 at a dose of 5 mg kg^–1^ was efficacious in decreasing hindlimb extension, while J4 did not decrease hindlimb extension until a higher dose (10 mg kg^–1^). Both JMF1907 and J4 were more potent in lengthening onset latency than ethosuximide (ETH) in PTZ-induced myoclonic epilepsy model, whereas ETH had better effects on the Racine score. In kindling model, JMF1907 and J4 at a dose of 1 mg kg^–1^ had effects on seizure frequency and duration, and the effects of JMF1907 were comparable with those of carbamazepine. Both JMF1907 and J4 can reduce the glutamatergic spontaneous excitatory post-synaptic currents (sEPSCs) frequency. The maximal inhibition was about 50% for JMF1907 at a concentration of 1 μg L^–1^, whereas J4 only inhibited 40% of sEPSCs frequency at a dose of 10 μg L^–1^.

**Conclusion and Implications:** ENT-1 inhibitors, JMF1907 and J4, showed anti-epileptic effects in different epilepsy models and the effects involved pre-synaptic neuronal modulation.

## Introduction

Epilepsy is a neurological disease characterized by the occurrence of transient paroxysms of excessive or uncontrolled discharges of neurons that leads to clinical manifestation of seizure. Despite that a number of antiepileptic drugs (AEDs) are currently available for clinical practice, about one third of patients with epilepsy remain refractory to the control of seizures no matter which AEDs is used alone or in combination (Kalilani et al., 2018). While currently used AEDs have been developed to suppress neuronal hyperexcitability, targeting pathogenic mechanisms involved in epilepsy development or progression may provide alternative therapeutic opportunities.

Adenosine is a well-characterized endogenous anticonvulsant. Extracellular levels of adenosine significantly increased by 6- to 31-fold in patients with intractable complex partial epilepsy during seizures (During and Spencer, 1992). Maladaptive changes in adenosine homeostasis were considered to occur during the pathogenesis of temporal lobe epilepsy (TLE) (Boison and Aronica, 2015). The elevated adenosine tone in the brain can terminate ongoing seizure activity or prevent the occurrence of seizure (Boison, 2005; Williams-Karnesky et al., 2013), particularly for pharmacoresistant epilepsy (Boison, 2007). On the other hand, an aberrant expression of adenosine A1 receptor whose activation is important for seizure suppression was identified in humans with epilepsy (Angelatou et al., 1993; Glass et al., 1996). In this regard, the dysregulation of adenosine-based neuromodulation may be associated with epileptogenesis and the augmentation of adenosine levels has been considered as a potentially therapeutic approach in epilepsy (Boison, 2012).

Extracellular adenosine level is controlled by a number of proteins. Extracellularly, ATP is converted to AMP and then to adenosine by CD39 and CD73, respectively. Intracellularly, adenosine is converted to inosine, S-adenosyl homocysteine, and AMP by adenosine deaminase, S-adenosyl homocysteine hydrolase, and adenosine kinase (ADK), respectively. It is generally believed that adenosine homeostasis in the brain is largely under the control of metabolic clearance through ADK, because the high affinity with adenosine, causing the inwardly net flux of adenosine through the nucleoside transporters located at the plasma membrane and maintaining the low extracellular adenosine level (Eltzschig et al., 2006; Boison, 2013). In terms of nucleoside transporters, the equilibrative nucleoside transporters (ENT), ENT-1, and ENT-2, are the major ones in the brain, responsible for the concentration-dependent influx or efflux of adenosine in the astrocytes and neurons. These transporters are known to be upregulated under some pathophysiological conditions such as ischemic and inflammatory via the hypoxia-inducible factor 1α (HIF-1α)-dependent signaling pathway (Poth et al., 2013; Bowser et al., 2017).

Indeed, an increase of ENT-1 expression was also identified in patients with epilepsy and in a pilocarpine-induced rat epileptic seizure model (Xu et al., 2015). Previously, JMF1907, an adenosine analog, has been demonstrated to be ENT-1 inhibitor that can increase brain extracellular adenosine levels in the striatum of mice by intraperitoneal administration and produces beneficial effects on transgenic mice with Huntington disease (HD) (Kao et al., 2017). Likewise, adenosine augmentation can be evoked by J4, another BBB-permeable adenosine analog and ENT-1 inhibitor, in the hippocampus and improves memory impairment and neuronal plasticity in the APP/PS1 mouse model of Alzheimer’s disease (AD) (Lee et al., 2018). Given that a deficiency in the homeostatic tone of adenosine may be a common pathophysiological mechanism for HD, AD, and epilepsy (Boison and Aronica, 2015), the objective of the present study was to examine whether JMF1907 and J4 produce beneficial effects on the treatment of epilepsy in animal models of maximal electroshock seizure (MES), and pentylenetetrazol (PTZ)-induced seizure and kindling models.

## Materials and Methods

### Animals

Male C57BL/6 (6–8 weeks old) mice were purchased from the Animal Center of the College of Medicine, National Taiwan University, and were housed in the animal quarters with air conditioning and on a 12-h light/dark cycle. Food and water were provided *ad libitum*. All animal treatments were conducted according to the Animal Research: Reporting In Vivo Experiments (ARRIVE) guidelines. The protocol of animal use was approved by the Institutional Animal Care and Use Committee of the National Taiwan University College of Medicine and College of Public Health.

### Electroencephalograms

Electroencephalograms (EEGs) studies were performed as previously reported (Tsai et al., 2015). Briefly, adult mice were anesthetized by intraperitoneal (i.p.) injection with ketamine/xylazine. Two EEGs electrodes were placed directly contact the left frontal and parietal lobes of cortices. The mice were then placed separately in individual recording cages and allowed to recover for 7 days before EEGs acquisition. The collected EEGs signals were amplified with an EEGs amplifier (V75-01, Coulbourn Instruments, Lehigh Valley, PA, United States) and band-pass filtered between 0.1 and 40 Hz. These conditioned signals were then subjected to analog/digital conversion (precision, 16-bit, sampling rate, 128 Hz) and stored in a personal computer for further analysis. Digitally stored EEGs data were visually scored offline using the AxoScope 10 software (Molecular Devices, Sunnyvale, CA, United States). The EEGs documented seizures were defined as the visualization of epileptiform spikes with amplitudes greater than 2 mV and lasting for at least 30 s.

### Mouse Seizure Models

#### Maximal Electroshock Seizure (MES) Model

In the electrically induced seizure experiments, mice were treated with vehicle, JMF1907 (0.05, 1, 5 mg kg^–1^) or J4 (1, 5, 10, 50 mg kg^–1^) 1 h prior to induction of convulsions. The MES was induced by a current generator (GEMORE Technology, New Taipei City, Taiwan). A fixed supramaximal stimulus current (80 mA, 0.3 s) was delivered in mice by way of saline-soaked corneal electrodes attached to both eyes. Seizure outcome was evaluated according to a modified Racine scale (Sakkaki et al., 2016): stage 0 (non-convulsive); stage 1 (immobility); stage 2 (mouth and facial movement); stage 3 (forelimb clonus and tail extension); stage 4 (rearing with forelimb clonus); stage 5 (generalized clonic seizure); stage 6 (rearing and falling with forelimb clonus followed by both forelimb and hindlimb extension). A suppression of hindlimb extension was considered as the positive measure of anticonvulsant activity.

#### PTZ-Induced Seizure Models

The mice were randomized into three groups. Group1 received normal saline and served as control. Group2 received the standard drug, ethosuximide (ETH) (150 mg kg^–1^). Group3 received the test drugs JMF1907 at a dose of 0.05, 1, and 5 mg kg^–1^ or J4 at a dose of 1 and 5 mg kg^–1^. PTZ (80 mg kg^–1^) was used to induce seizure 1 h after the saline, ETH, or test drugs treatment. Mice were observed for 20 min after injection of PTZ. Protection against PTZ-induced seizure and death in mice was determined.

#### PTZ-Induced Kindling Models

The PTZ kindling epilepsy model was induced as previously described (Yi et al., 2016). Briefly, the EEGs electrode-implanted mice were injected with a subconvulsive dose of PTZ (35 mg kg^–1^) on 1-day interval between each injection. According to the methods reported by Yi et al. (2016), five injections of PTZ were required to acquire kindling. The kindled seizures were considered successful when the spontaneous epileptic waveforms on hippocampus EEGs (as defined above) were observed. Successfully kindled animals were then injected with test drugs (CBZ, carbamazepine, 4 mg kg^–1^, JMF1907, 5 mg kg^–1^, and J4, 5 mg kg^–1^) per day for 1 week. Seizures activities (i.e., seizure duration and rate of occurrence) were evaluated.

### Electrophysiology

Electrophysiological studies were performed as previously reported (Ho et al., 2012). Briefly, the mice were anesthetized via isoflurane inhalation and decapitated. The brains were rapidly removed and placed in ice-cold (0–4°C) cutting solution containing (in mM) : 0.5 CaCl_2_, 110 choline chloride, 25 glucose, 2 KCl, 1.25 NaH_2_PO_4_, 7 MgSO_4_, 26 NaHCO_3_, 11.6 sodium ascorbate and 3.1 sodium pyruvate. Coronal brain slices (300∼350 μm thick) containing hippocampus were cut using a microslicer (DTK-1000, Dosaka, Japan) and then transferred to a recovery chamber with artificial cerebrospinal fluid (aCSF) consisting (in mM) : 2 CaCl_2_, 10 glucose, 3KCl, 1 MgCl_2_, 125 NaCl, 26 NaHCO_3_ and 1.25 NaH_2_PO_4_. Slices were kept for 1 h at room temperature in aCSF bubbled with carbogen (95% O_2_ and 5% CO_2_), pH 7.3–7.4. After recovery, the brain slices were then transferred to a recording chamber perfused with aCSF at 1–2 ml/min. Whole-cell recordings were made from the hippocampal dentate granule cells by using a patch-clamp amplifier (Axopatch 200B, Molecular Devices, United States). Neurons were visualized under the infrared-DIC camera by an upright microscope (BX51WI, Olympus, Japan). Patch electrodes were pulled from borosilicate glass capillaries to tip resistances of approximately 5–8 MΩ by a micropipette puller (P97, Sutter Instrument, United States). The electrodes were filled with a K-gluconate (K-Glu) based internal solution containing (in mM) : 140 K-Glu, 5 KCl, 10 HEPES, 0.2 EGTA, 2 MgCl_2_, 4 MgATP, 0.3 Na_2_GTP and 10 Na_2_-phosphocreatine, pH 7.2 (with KOH). Data acquisition was performed by using a digitizer and pClamp 10 software (DigiData 1440A, Molecular Devices, United States). To isolate the AMPA receptor-mediated spontaneous excitatory post-synaptic currents (sEPSCs), the cell was voltage-clamped at −70 mV in the presence of a GABA receptor antagonist picrotoxin (100 μM). Signals were filtered at 2 Hz and digitized at 10 Hz. Quantifications were performed on 5 min segments after 10–20 min of drug treatment and compared to the average of 5 min of control period. Data were detected and analyzed using MiniAnalysis Program (Synaptosoft, United States). Detection parameters were set at >5 pA amplitude, <1 ms rise time, and <3 ms decay time.

### *In vivo* Brain Microdialysis and Measurements of Adenosine

The microdialysis experiment was performed according to the methods described by Lee et al. (2018). Mice were anesthetized by i.p. injection with ketamine/xylazine and fixed on a stereotaxic instrument (Stoelting, Wood Dale, IL, United States). A vertical guide cannula was stereotaxically implanted into the hippocampus (anteroposterior, ±2.8 mm; mediolateral, ±3.0 mm; dorsoventral, −2.3 mm). After 3 days cannulation, a microdialysis probe (MAB 10.8.2.Cu, Microbiotech/se AB, Stockholm, Swedish) was inserted into the mouse brain through the guide cannula and infused with Ringer’s solution (1 μl/min) for 4 h. The brain outflow was collected every 30 min. The samples were frozen at −20°C until assayed. For adenosine measurements, the adenosine was first converted to fluorescent 1,N^6^-etheno-adenine derivatives. The supernatant was then injected into an HPLC system (Hitachi, Tokyo, Japan) and tested using a COSMOSIL 5C18-AR-II column (5 μm, 250 × 4.6 mm, Nacalai Tesque, Inc., Kyoto, Japan) equipped with a C18 SecurityGuard cartridge (Phenomenex, Torrance, CA, United States).

### Statistical Analysis

Data were analyzed by Student’s *t*-test, Fisher exact test or two-way ANOVA followed by Fisher’s LSD test (unless otherwise noted) using SYSTAT (version 12, SYSTAT software, Inc., Chicago, IL, United States). A level of significance of 0.05 was considered statistically significant.

## Results

### Effects of JMF1907 and J4 on MES

**FIGURE 1:**
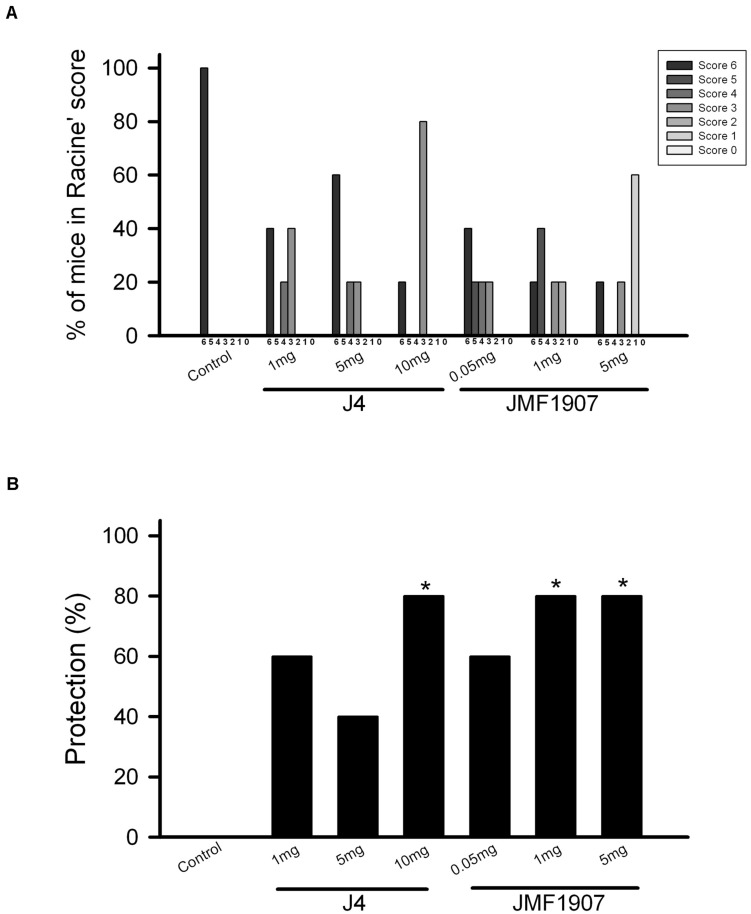
Effects of JMF1907 and J4 on seizure control induced by maximal electroshock. Adapted Racine score was measured for all groups **(A)**. The proportion of mice against MES-induced hindlimb extension (score 6) in each group **(B)**. The efficacy of the drug treatment was evaluated to the response obtained from the control group by using the Fisher exact test. The asterisk indicates *p* < 0.05 compared to controls.

The maximal electroshock model has been used to generate the generalized tonic-clonic seizure. To evaluate the effects of test compounds on seizure control in tonic-clonic model, JMF1907 (0.05, 1, 5 mg kg^–1^) and J4 (1, 5, 10 mg kg^–1^) were given by i.p. administration 1 h before the induction of MES. The animal exposed to MES developed seizures were classified according to an adapted Racine’s scale (Figure 1A). The results showed that the tonic hindlimb extension (score 6) was significantly reduced (by 80%) in mice treated with JMF1907 at doses 1 and 5 mg kg^–1^, compared to the control group. On the other hand, the treatment of J4 did not significantly decrease the hindlimb extension until it was given at a dose of 10 mg kg^–1^ (Figure 1B). These findings suggest that the effect of JMF1907 in MES is better than J4.

### Effects of JMF1907 and J4 on Seizure Induced by High Dose PTZ Treatment

**FIGURE 2:**
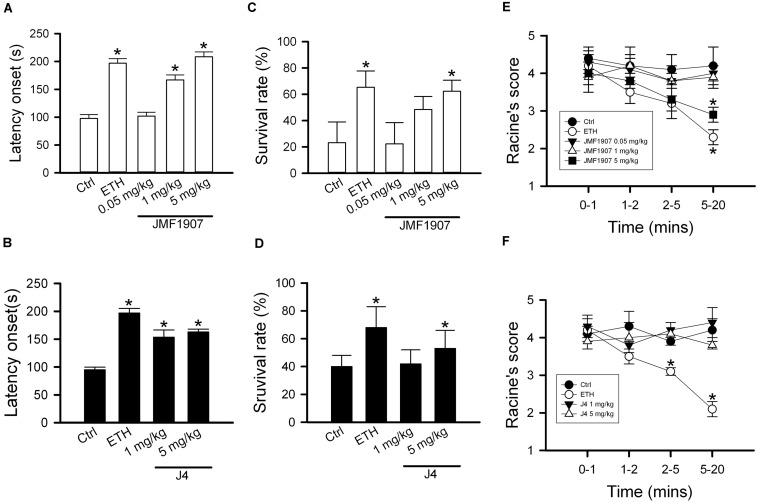
Effects of JMF1907 **(A,C,E)** and J4 **(B,D,F)** on seizure induced by high dose PTZ in B6 mice. Ethosuximide (150 mg/kg) was used as a reference. Data are given as mean ± SEM of five animals. The asterisk indicates *p* < 0.05 (Student’s *t*-test) compared to controls.

Pentylenetetrazol-induced seizure is considered as a model for generalized myoclonic seizure. To examine the effects of test compounds on this seizure model, JMF1907 (0.05, 1, 5 mg kg^–1^) and J4 (0.05, 1, 5 mg kg^–1^) were given 1 h before i.p. administration of PTZ. As ETH can be used to control myoclonic seizure, it was used as a positive control. The mean latency to seizure onset was 97.6 ± 7.2 s in control group after PTZ induction. ETH significantly prolonged the onset of seizure (197.0 ± 8.2 s, *p* < 0.05) at dose 150 mg kg^–1^. Pre-treatment of JMF1907 remarkably extended the onset latency (167.0 ± 8.9 s and 208.4 ± 8.9 s, respectively, *p* < 0.05) in PTZ-induced seizure at doses 1 and 5 mg kg^–1^, respectively (Figure 2A). Likewise, the treatment of J4 also significantly delayed seizure occurrence following PTZ (95.0 ± 4.9 s for controls; 153.6 ± 12.9 s for 1 mg kg^–1^; 163.0 ± 4.9 s for 5 mg kg^–1^; *p* < 0.05, Figure 2B). Furthermore, our results showed that JMF1907 and J4 at a dose of 5 mg kg^–1^ significantly increased the percentage of survival after PTZ administration (JMF1907 group, ctrl, 23.2 ± 15.7%, 5 mg kg^–1^, 62.3 ± 8.4%; J4 group, ctrl, 40.0 ± 8.0%, 5 mg kg^–1^, 53.0 ± 13.0%, *p* < 0.05, Figures 2C,D). Interestingly, in seizure severity analysis (based on Racine scale), we found that only JMF1907 at 5 mg kg^–1^ dosage can reduce severity levels during the observation period and displayed a similar effect as ETH (Figures 2E,F, *p* < 0.05). These results indicate both JMF1907 and J4 produced beneficial effects on seizure control, in which the action of JMF1907 is a dose-dependent manner and more potent in the suppression of myoclonic seizure.

### Effects of JMF1907 and J4 on PTZ-Induced Kindling Seizure Model

**FIGURE 3:**
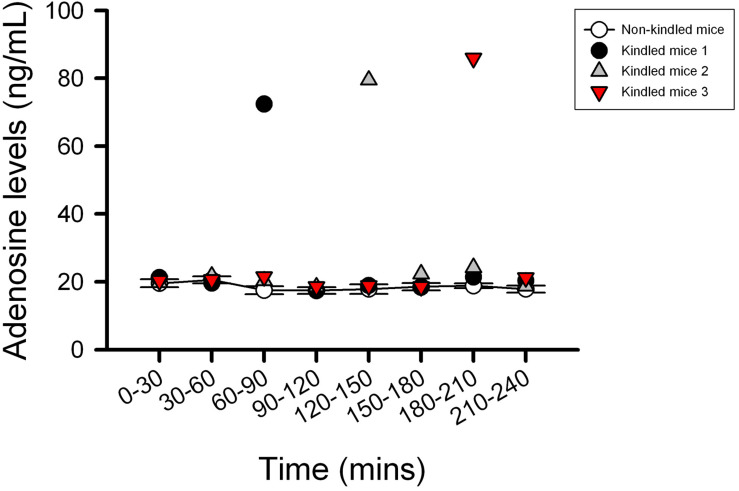
Characterization of the extracellular adenosine level in kindled mice hippocampus by microdialysis. The kindled mice showed dynamic elevated hippocampus levels of adenosine in the range of 72–85 ng/ml during the sampling period. In contrast to the kindled mice, the averages of extracellular adenosine concentrations in non-kindled mice were about 20 ng/ml and showed no significant change in adenosine levels for 4 h. Data are given as mean ± SEM of six animals (non-kindled group).

**FIGURE 4:**
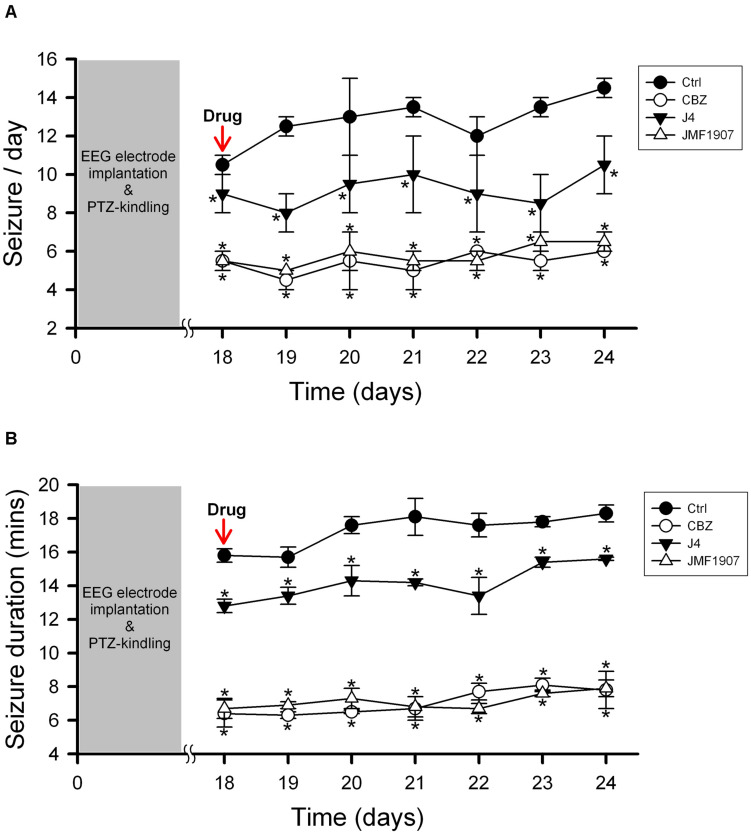
Effects of JMF1907 and J4 on occurrence of seizure per day **(A)** and seizure duration **(B)** in the PTZ-induced kindling seizure model. Carbamazepine (CBZ, 4 mg kg^– 1^) was used as a reference. Data are given as mean ± SEM of five animals. Statistical analyses were calculated using the repeated measures two-way ANOVA followed by Fisher’s LSD test. Within days significances: * *p* < 0.05 compared to Ctrl group.

The kindling model produced by repetitive low dose (35 mg kg^–1^) PTZ treatment is a representative model for partial seizure. In our study, five injections of PTZ were required to acquire kindling, as indicated by progressive increase in the spontaneous seizure frequency. Notably, with microdialysis studies, we observed the extracellular levels of adenosine in kindled-mice hippocampus were dynamically increased during the sampling periods (Figure 3), similar to the previous report on Sprague-Dawley rats (Aden et al., 2004). In this model, CBZ was used as a reference control. JMF1907 treatment produced beneficial effects in reducing the occurrence of seizure per day and seizure duration at a dose of 1 mg kg^–1^, which were comparable to that of CBZ (4 mg kg^–1^). Although the J4 (1 mg kg^–1^) also improved the seizures activity in comparison to control group, the protective effect of J4 was significantly lower than CBZ and JMF1907. The average seizure number and duration per day during the period of drugs treatment were as followed : ctrl, 10.5 ± 0.5–14.5 ± 0.5/15.7 ± 0.6–18.3 ± 0.5 min, CBZ, 4.5 ± 0.5–6.0 ± 0.0/6.1 ± 0.5–8.1 ± 0.4 min, JMF1907, 5.0 ± 0.0–7.5 ± 1.0/6.7 ± 0.6–7.9 ± 0.5 min, J4, 8.0 ± 1.0–10.5 ± 1.5/12.2 ± 0.1–15.6 ± 0.1 min, Figures 4A,B).

### Effects of JMF1907 and J4 on sEPSCs Frequency

**FIGURE 5:**
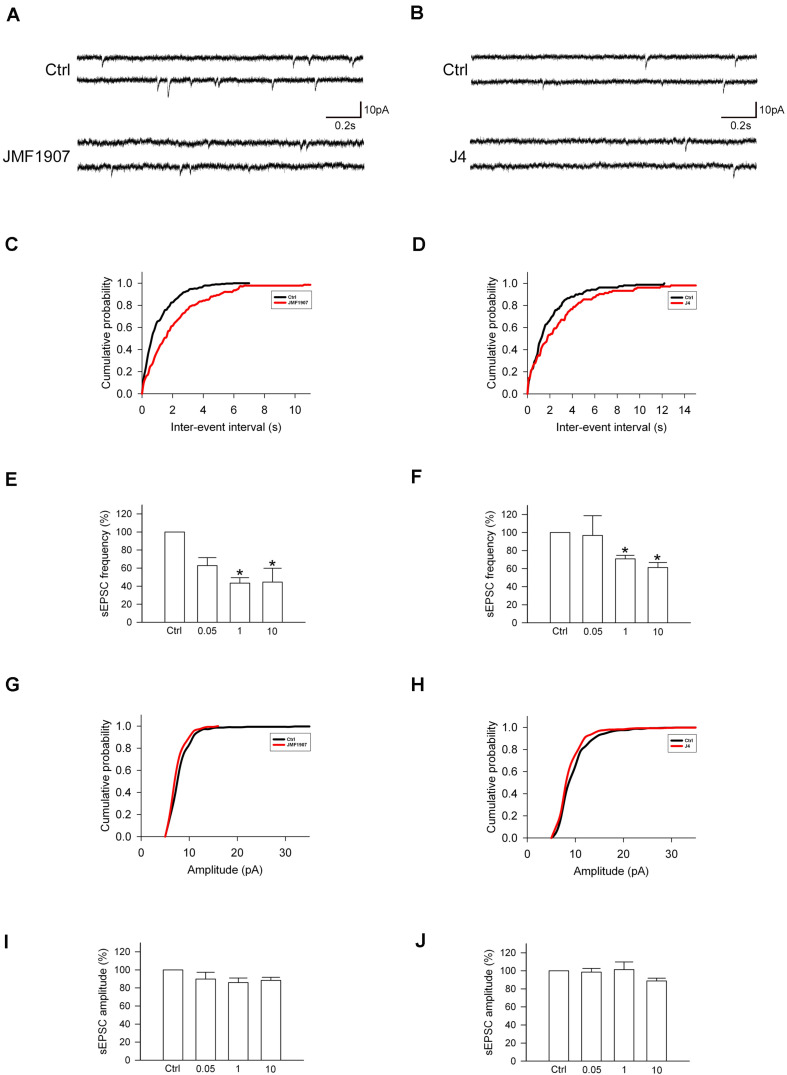
Effects of JMF1907 and J4 on sEPSCs recorded from hippocampal granule cells. **(A,B)** Sample traces of sEPSCs from wild-type mice before and after 1 μg L^– 1^ JMF1907 and J4 perfusion. The cumulative histograms were obtained from the same neurons illustrated in panel **(A,B)** before and after drugs treatment. The result showed a decrease in sEPSCs frequency (**C,D**, as measured by the inter-event intervals, *p* < 0.0001) but not amplitude (**G**, *p* = 0.54; **H**, *p* = 0.66, Kolmogorov–Smirnov test) after JMF1907 and J4 treatment. **(E,F,I,J)** Changes in the average frequency and amplitude of sEPSCs after 0.05-, 1-, and 10 μg L^– 1^ JMF1907 and J4 application. These results indicate that both JMF1907 and J4 can reduce the sEPSCs frequency in a dose-dependent manner. Data are given as mean ± SEM (*n* = 3–5 neurons per group obtained from 2 to 3 animals). Statistical significance (*p < 0.05) was evaluated with Student’s *t*–test.

Glutamate is a major excitatory neurotransmitter that plays a critical role in the seizure generation and propagation. To examine the acute effects of JMF1907 and J4 on the glutamatergic neurotransmission, we performed *in vitro* whole-cell patch-clamp recording from dentate gyrus granule cells in brain slices obtained from wild-type adult mice (Figures 5A,B). The results showed that both JMF1907 and J4 can reduce the frequency (Figures 5C–F) but not the amplitude (Figures 5G–J) of sEPSCs. The maximal inhibition was about 50% for JMF1907 at a concentration of 1 μg mL^–1^ (2.4 μM) and was about 40% inhibition for J4 at a concentration of 10 μg mL^–1^ (23.7 μM). These results suggest that the JMF1907 or J4 exhibit anticonvulsant effects possibly mediated via the glutamatergic pathway.

## Discussion

There has been a concern that AEDs development is struggling to discover compounds that are distinct from current medications. It is also challenging to have new drugs that significantly change epilepsy prognosis, especially for patients with intractable epilepsy. While the mechanisms for drug resistance seizure remain elusive, it is related to the characteristics of epilepsy, pharmacological mechanisms of resistance due to overexpression of drug efflux transporters, and loss of AEDs sensitivity (Santulli et al., 2016). It is recognized that the disruption of adenosine homeostasis plays a major role in epileptogenesis and the reconstruction of adenosine functions may provide a way for the prevention of epileptogenesis (Boison, 2013,2016; Cieslak et al., 2017). In line with this concept, local delivery of adenosine through polymers implanted into the brain has been proved to be efficacious for seizure control in kindled rats (Szybala et al., 2009). To be non-invasive, alternatively, earlier studies have described the beneficial effect of adenosine transport inhibitors on seizure suppression *in vitro* (Kostopoulos et al., 1989) and *in vivo* (Zhang et al., 1993).

In the present study, we demonstrated that i.p. administrated BBB-permeable ENT-1 inhibitors, JMF1907 and J4, can produce beneficial effects in various seizure models, including seizure induced by MES, high dose PTZ, and low dose PTZ kindling that represent generalized tonic-clonic seizure, generalized myoclonic seizure, and focal seizure, respectively. Adenosine has effects both on seizure generation (ictogenesis) and development (epileptogenesis) (Boison, 2016). In MES model, adenosine antagonist reduced post-ictal depression (Kulkarni et al., 1994). Adenosine A1 receptors (A1Rs) agonist also showed antiepileptic effects in the mouse MES-induced seizure model (Luszczki et al., 2005). Other studies have shown decreased density of A1Rs in the PTZ-induced seizures (Cremer et al., 2009) and kindled seizure (Rebola et al., 2003). In zebrafish PTZ-induced seizure model, both the A1Rs agonist cyclopentyladenosine (CPA) and ENT-1 inhibitor nitrobenzylthioinosine (NBTI) increased the latency to reach the tonic-clonic seizure stage (Siebel et al., 2015).

It has been known that expression of hippocampal ENT-1 was elevated in patients with TLE and rat model of pilocarpine-induced epilepsy, while intrahippocampal injection of NBTI, can attenuate seizure severity and prolongs onset latency in pilocarpine-induced seizure in rats (Xu et al., 2015). In contrast, single nucleotide polymorphism study of human ENT-1 gene (*SLC29A1*) showed T647C variant would increase risk of alcohol withdrawal seizure, along with decreased extracellular adenosine level (Kim et al., 2011). As J4 has been shown to increase extracellular adenosine level (Lee et al., 2018), adenosine augmentation caused by ENT-1 inhibition may play a major role in reduction of seizure activity.

The different purinergic mechanisms controlling glutamate release are depending on the mechanism of neurotransmitter release (for example, evoked versus spontaneous). Previous studies demonstrated that an elevation of adenosine in CA1 pyramidal neurons caused adenosine release into the extracellular space and therefore selectively inhibited evoked glutamatergic EPSC (Brundege and Dunwiddie, 1996). In our study, though the level of evoked EPSCs was not validated, we found significantly decreased sEPSCs (action potential-dependent release of neurotransmitter) frequency but not amplitude after ENT-1 inhibitors were applied. These data revealed the site of action for JMF 1907 and J4 on reducing excitatory neuronal activities pre-synaptically. Xu et al. (2015) found that reduction of miniature EPSCs (action potential-independent release of neurotransmitter) frequency but not amplitude after application of NBTI in pilocarpine-induced rat seizure model. Indeed, transgenic mice expression human ENT-1 resulted in decrease of basal pre-synaptic A1Rs function (Zhang et al., 2011), and adenosine release during hypoxia induces a decrease in pre-synaptic glutamate release in area of hippocampal CA3 (Jarosch et al., 2015). Adenosine has been shown to reduce frequency of action potential-independent mEPSPs (miniature excitatory post-synaptic potentials) by the activation of pre-synaptic A1Rs, which suppresses excitatory transmission by reducing release probability (Bannon et al., 2014). These results may suggest ENT-1 inhibitor acts on modulation of adenosine level, which may influence pre-synaptic neurons (action potential-dependent and independent) consequently.

In fact, adenosine level rises as a consequence of seizures and is regarded as an endogenous protective mechanism for epilepsy (Hargus et al., 2012). The elevated extracellular adenosine can be transported into astrocyte via ENT-1, and is later metabolized by intracellular ADK, causing the inwardly net flux of adenosine and maintaining the low extracellular adenosine level (Boison, 2013). In mesial TLE induced by kainic acid, ADK expression was initially down-regulated but then up-regulated during the progression of epileptogenesis (Gouder et al., 2004). Given that an elevation of extracellular adenosine produces beneficial effects on seizure control, the development of ADK inhibitors seems to a feasible way for the treatment of epilepsy. However, it is noted that the systemic application of ADK inhibitors can be associated with a number of adverse reaction, including a decrease of cardiovascular functions and the development of hepatic steatosis (Wiesner et al., 1999; Boison et al., 2002; Gouder et al., 2004). As an upstream mediator of adenosine degradation, ENT-1 is a potential candidate to avoid subsequent epileptogenesis. While the safety of using ENT-1 inhibitors needs to be examined. Although clinical use of dipyridamole, as an ENT-1/ENT-2 inhibitor, appears to be safe (De Schryver et al., 2006), dipyridamole is not BBB-permeable.

In search for new AEDs, especially for refractory epilepsy, the US FDA has recently approved a drug made from cannabidiol for the treatment of two rare and serious forms of epilepsy, Lennox-Gastaut syndrome and Dravet syndrome. While cannabidiol might influence neuronal hyperexcitability by several mechanisms (Ibeas Bih et al., 2015), the molecular targets by which cannabidiol exerts its anti-seizure activity are still undetermined. Nevertheless, the antiepileptogenic effects of cannabidiol were shown to be independent of the endocannabinoid-signaling pathway (Rosenberg et al., 2017) and may be related to its effect on the ENTs (Devinsky et al., 2014). While systemic administration of adenosine is not feasible because of several cardiovascular adverse effects, the delivery of adenosine locally into the brain is invasive. In accordance with this, the enhancement of adenosine signaling through the inhibition of ENT-1 is also considered to be an important non-cannabinoid receptor mechanism for the immunosuppressive effects of Cannabidiol (Carrier et al., 2006). The inhibition of ENT-1/ENT-2 has also been shown to produce beneficial effects on the animal models of HD (Kao et al., 2017) and AD (Lee et al., 2018), stress echocardiography (Koeppen et al., 2009), mucosal inflammation (Aherne et al., 2018), and acute lung injury (Eckle et al., 2013). These findings strengthen the importance of adenosine tone in brain and other organ functions and the potential of developing ENT-1 inhibitors for the treatment of epilepsy as well as for other brain disorders.

## Conclusion

In conclusion, the disruption of adenosine homeostasis is important for the epileptogenesis and the augmentation of adenosine levels can be considered as a potentially therapeutic approach in epilepsy. Systemic administration of JMF1907 and J4 produces beneficial effects on the treatment of epilepsy in acute and chronic seizure models. Given that ENT-1 is an important for cellular uptake of adenosine in the brain, the development of ENT-1 inhibitors for the treatment of epilepsy is worth an attention.

## Data Availability Statement

The raw data supporting the conclusions of this article will be made available by the authors, without undue reservation.

## Ethics Statement

The animal study was reviewed and approved by Institutional Animal Care and Use Committee, National Taiwan University.

## Author Contributions

S-YH: writing, electrophysiology. K-CC: writing. I-CC, C-WT, and H-RL: behavior test. C-JL and H-HL: writing, principle investigator. All authors contributed to the article and approved the submitted version.

## Conflict of Interest

The authors declare that the research was conducted in the absence of any commercial or financial relationships that could be construed as a potential conflict of interest.
